# Two-leaves and many bites: Profiling dog-bites and adherence to rabies prophylaxis in tea-estate communities of Udalguri District, Assam, India

**DOI:** 10.1371/journal.pntd.0013791

**Published:** 2025-12-01

**Authors:** Harish Kumar Tiwari, Parimala Mohanty, Rasika G. Shirke, Aina Unnikrishnan Kurup, Chalasani Satwik, Shanti Priya Kindo, Riya Shigwan, Karma Wangdi, Jully Gogoi-Tiwari, Laura Cunha Silva, Salome Dürr

**Affiliations:** 1 Jyoti and Bhupat Mehta School of Health Science and Technology, Indian Institute of Technology Guwahati, Guwahati, Assam, India; 2 JH CreIndia Foundation, Guwahati, Assam, India; 3 DBT-Wellcome Trust India Alliance Intermediate Fellow, Hyderabad, Telangana, India; 4 DY Patil University School of Public Health, Navi Mumbai, Maharashtra, India; 5 Indian Institute of Public Health Delhi- Delhi, Public Health Foundation of India, Gurgaon, Haryana, India; 6 School of Veterinary Medicine, College of Agricultural and Life Sciences, Murdoch University, Murdoch, Western Australia, Australia; 7 Veterinary Public Health Institute, Vetsuisse Faculty, University of Bern, Bern, Switzerland; Colorado State University, UNITED STATES OF AMERICA

## Abstract

**Background:**

Dog-mediated rabies disproportionately affects marginalised and socioeconomically disadvantaged communities. Tea estate (TE) communities in India exemplify one such vulnerable population. Despite their vulnerability, limited research has explored rabies epidemiology within TE settings. This retrospective study uses secondary data to evaluate the incidence of dog bite and their determinants amongst the TE communities in the Udalguri district of Assam state of India.

**Methods:**

Secondary data from 17 to 29 months (January 2022 to May 2024) were retrieved from the hospitals and dispensaries of 11 TE of Udalguri district, Assam. The collected information included dog-bite victims’ demographics and adherence to post-exposure prophylaxis (PEP). Data were analysed using R software, employing descriptive statistics, chi-square tests, odds ratios and mixed-effect logistic regression. Administrative approval was obtained prior to data collection.

**Results:**

A cumulative annual incidence of 11.8 bites per 1,000 individuals was recorded across 11 TE in Udalguri. Children aged ≤15 years accounted for 35% of cases, and dependents were the most affected occupational group (32%). Most exposures involved dogs (66%), and 76% of incidents were bites. Less than half (43%) of victims completed the full PEP regimen of five doses, although 71% received at least three doses. Chi-square analysis indicated that males and children aged ≤ 15 years were more likely to be bitten by dogs compared to other animals than females and the older residents. Children aged ≤ 15 years and non-workers had higher odds of receiving any PEP, while females and children aged ≤ 15 years are more likely to receive at least three doses. In multivariable analysis, females were less likely than males to be bitten by dogs compared to other animals (aOR = 0.4, 95% CI: 0.3–0.7), older individuals had higher odds of completing PEP (aOR = 1.8, 95% CI: 1.2–2.8), and children (≤15 years) were more likely to receive at least three doses of PEP (aOR = 1.9, 95% CI: 1.1–3.3). Temporal analysis showed no clear seasonal pattern, although spikes were observed during winter and monsoon months.

**Conclusion:**

This retrospective study contributes to build the foundation for community-based approach to control dog-mediated rabies in TE by highlighting key epidemiological patterns, demographic vulnerabilities and limitations of the existing intervention implementation delivery among TE communities. We recommend further in-depth investigations to inform the context specific interventions designed to address the unique vulnerabilities, thereby reducing the risk of rabies specifically in tea -estate populations.

## Author summary

This retrospective study analysed secondary data of hospital and dispensary records from 11 tea estates in Assam’s Udalguri district between January 2022 and May 2024 to estimate the incidence of dog bites, profile affected individuals, and assess access to and completion of post-exposure prophylaxis (PEP). The findings bring to light the gaps in rabies prevention, particularly in remote areas and underserved tea -estate communities, where delayed or incomplete PEP administration significantly increases the risk of rabies-related fatalities. The study highlights the pressing need to expand rabies surveillance to include marginalised populations, strengthen health systems in remote areas, improve PEP availability and adherence, and design community-specific strategies. By identifying barriers to timely and complete adherence to PEP, this study provides actionable insights such as expanding the research base to include vulnerable communities such as tea-estate communities, developing tailormade strategies, and better adherence to post-bite prophylaxis to guide policy-making and the implementation of targeted interventions to reduce the burden of rabies in such high-risk populations.

## 1. Introduction

Rabies is endemic to India, with approximately 99% of human rabies deaths resulting from dog bites [1]. The disease disproportionately affects marginalised and socioeconomically disadvantaged populations who often lack awareness of the consequences of untreated dog bites and have limited capacity to seek timely medical care [2–5]. Moreover, free-roaming dogs (FRD) often reside near the residential settlements of such vulnerable communities, significantly increasing the risk of dog bites [2,6–9]. Post-exposure prophylaxis (PEP) is the only effective practice to prevent the onset of rabies following a suspected exposure, provided it is administered promptly and appropriately [10]. However, the lack of availability, difficult accessibility, and poor affordability of PEP accentuate the challenging circumstances, leading to mortality [11]. These challenges get pronounced in remote and underserved regions, where health infrastructure is often inadequate or absent, leaving vulnerable communities without access to critical care [12,13].

Several countries have developed action plans for eliminating dog-mediated rabies in response to the ‘Zero by 30’ campaign, initiated under the tripartite agreement between WHO (World Health Organization), FAO (Food and Agriculture Organization), and the WOAH (World Organisation of Animal Health). While many of these plans address the disease in vulnerable populations, few explicitly identify or define these communities.

Addressing dog-mediated rabies in vulnerable populations requires confronting the broader social inequities in healthcare access before developing strategies that can be equitably scaled at the national level [14]. However, one of the major impediments to the judicious allocation of resources for implementing interventions for rabies control is the lack of reliable surveillance data, especially from the most affected communities [15,16]. While population-based data on rabies in humans and FRD provide some insight into communities’ attitude about the disease and FRD, a differentiated policy assessment grounded in principles of health equity is essential for designing context-sensitive interventions that respond to the diverse needs of affected populations [17].

Assam, located in northeast India, is one of the country’s major tea-producing states where workers are employed in various capacities, including labour-intensive roles such as manually plucking tea leaves [18]. The tea estates (TE) are usually remotely located, and in many instances, far from accessible health centres equipped with adequate facilities [19]. However, some TE do have health centres for workers within the estate. Tea -estate workers are predominantly from socioeconomically disadvantaged backgrounds, many of whom are illiterate and work under gruelling circumstances that increase their vulnerability to animal attacks, including dog bites [20]. Despite representing a significant public health risk in these remote communities, the epidemiology of dog bites and rabies remains poorly documented. It is important to bridge such knowledge gaps to institute informed and targeted interventions by analysing existing data and adopting a spatial approach instead of a generalised one [17,20–22].

This study explores the epidemiology of dog bites and adherence to rabies prophylaxis in tea estate communities of the Udalguri district, Assam, India, using secondary data. The study hypothesized that:

(a) Children and dependents have higher incidence of dog-bites compared to adults and workers
(b) Females and children are less likely to complete the full regimen than males and older residents
(c) Access to and completion of PEP vary across demographic and occupational groups within the tea-estate communities.

The study is guided by the above-mentioned hypotheses to evaluate dog-bite incidence, profile at risk populations, and assessment of PEP accessibility and adherence.

## 2. Materials and methods

**Ethics statement:** The study was approved by the Institutional Human Ethics Committee (IHEC) of the Indian Institute of Technology Guwahati (IHEC/2024/03). Administrative approval for data collection was obtained from the respective TE authorities before commencing the study.

### 2.1. Study area and approvals

**Fig 1:**
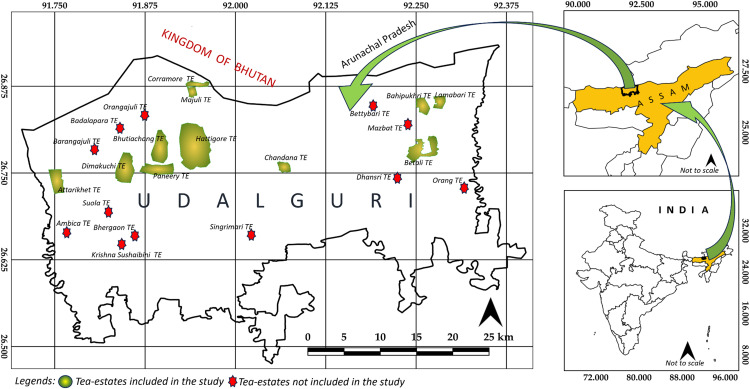
Locations of the Tea-Estates of the Udalguri district in Assam, India from where secondary data of dog-bites analysed in the study was collected (Base map for ‘India’ and ‘State boundaries’ sourced from https://simplemaps.com/gis/country/in and ‘District boundaries obtained from https://projects.datameet.org/maps/districts).

This study was conducted in the Udalguri district in the North Assam division of Assam, northeast India (26.7452°N and 92.0962°E). The district shares an international border with Bhutan and a state boundary with Arunachal Pradesh (Fig 1). Udalguri is home to 22 TE, of which 11 consented to participate. These estates vary in size and workforce composition and are representative of the district’s tea-producing landscape.

### 2.2. Data collection

Secondary data were retrieved from TE dispensaries and hospital records from January 2022 to May 2024. Although these records were maintained in the dog-bite registers but also included bites from other animals suspected of transmitting rabies (cats, rats, or mongooses). Data were manually extracted from hardcopy registers and entered into Microsoft Excel. The collected data included the demographics of the bite victims, details of PEP, species of the biting animals, and the location of bites. Identifying information such as names and address of the bite victims were excluded. Three TEs maintained data from January 2022 (29 months), while eight provided records from January 2023 (17 months). The dataset was deemed adequate for the planned analyses.

### 2.3. Inclusion criteria and variable classification

Data entries containing at least two explanatory variables and three outcome variables were included for the regression analysis. Explanatory variables comprised age, gender, and employment status; outcome variables included adherence to the PEP regimen (complete/incomplete), the number of doses administered (≥3/ < 3), and species of the biting animal (dog/others). Age was categorised into five groups: ≤ 15 years, 16–30 years, 31–45 years, 46–60 years and >60 years. Employment status comprised ‘permanent workers’, ‘temporary workers’, ‘non-workers’ (adult family members or visitors not directly employed), and ‘dependents’ (family members ≤ 18 years). Average monthly temperature and relative humidity for the study period were obtained from Climate & Weather Averages in Udalguri, Assam, India [23].

### 2.4. Data analysis

Dog-bite incidence was calculated as the number of bites per 1,000 individuals per year. Chi-square goodness-of-fit tests compared observed and expected bite incidences for each explanatory variable. Chi-square tests of independence and corresponding odds ratios were used to examine associations between explanatory and outcome variables.

Three mixed-effects logistic regression analyses were conducted—each for one outcome variable (biting species, completion of PEP, and receipt of ≥ 3 doses)—with tea estate as a random effect to account for clustering. The model diagnostics were assessed through stepwise comparison of the mixed-effect models with generalised linear models (GLMs) with the same outcome and explanatory variables, using Akaike Information Criterion (AIC), Type-II Wald chi-square tests, and marginal and conditional R^2^ estimates. A Poisson regression model evaluated the effect of employment status on the bite incidences. Correlations between monthly dog-bite counts and average temperature and humidity were tested using Pearson’s correlation coefficient. All analyses were performed in R (version 4.3 or later) using the packages *epiR*, *epitools*, *tidyverse*, *lme4, ggplot2*, *DHARMa*, *MuMIn*, and *patchwork* [24–32].

## 3. Results

### 3.1. Incidence of dog-bites

Across the 11 TEs, a total of 1,187 bite cases were recorded. Annual incidence rates ranged from 1.5 to 36.9 bites/1000 individuals across the 11 TEs, with Hattigore TE recording the highest, and Betali TE the least (Table 1). Permanent staff and the dependents recorded the highest incidence (19.8/1000 individuals per year) while the temporary staff recorded the lowest.

**Table 1:** Details of the bite incidence calculated from the secondary data (01 January 2022- 31 May 2024) including bites in each occupational category obtained from 11 tea-estates in the Udalguri District of Assam, India.

S. no.	Tea-Estates	Missing data*	Permanent staff (number of bites)	Temporary staff (number of bites)	Dependents (number of bites)	Non-workers (number of bites)	Total staff (number of bites)	Annual incidence (number of bites/1000 individuals)
1	Atterakhat^#^	–	770	100	842	1518 (8)	3230 (8)	**1.7 (0.8 -3.4)**
2	Bhutiachang^#^	39	1022 (7)	799 (1)	1032 (10)	620	3473 (57)	**11.6 (8.8 -15.0)**
3	Bahipookhri^#^	23	858	1285	1646	797	4586 (29)	**4.5 (3.0-6.4)**
4	Betali^#^	–	568 (2)	1460	2272 (3)	817 (6)	5117 (11)	**1.5 (0.8 -2.7)**
5	Chandana^#^	6	355	40	490 (3)	400	1285 (9)	**4.9 (2.3 -9.4)**
6	Corramore^#^	4	829 (20)	678	734 (31)	326	2567 (55)	**15.1 (11.4 -19.7)**
7	Lamabari^#^	1	702 (6)	456	367 (28)	2322	3847 (35)	**6.4 (4.5 -8.9)**
8	Dimakuchi^#^	49	759	510	776 (45)	459	2504 (94)	**26.5 (21.4 -32.4)**
9	Hattigore**	0	1535 (232)	1828 (38)	930 (219)	4250 (271)	8543 (762)	**36.9 (34.3 -39.6)**
10	Majuli**	1	738 (13)	810 (5)	534 (6)	1753 (8)	3835 (33)	**3.6 (2.5 -5.0)**
11	Paneery**	45	347 (10)	461 (3)	876 (2)	956 (14)	2640 (94)	**14.7 (11.9 - 18.0)**
**Total**		**168**	**8483 (290)**	**8427 (47)**	**10499 (375)**	**14218 (307)**	**41627 (1187)**	**16.0 (15.1 - 17.0)**
**Annual incidence (number of bites/1000 individuals)**			**19.8 (17.6-22.2)**	**3.1 (2.3-4.2)**	**19.8 (17.7 -22.0)**	**11.3 (10.1-12.7)**	**16.0 (15.1 -17.0)**	

*Bite records did not contain individual’s demographic details; # records available only for 17 months; ** records available for 29 months.

### 3.2. Demographic and occupational distribution of bite victims and completion of PEP

Among children aged ≤ 15 years, 160 (38%) were ≤ 5 years, including one 9-month-old infant. Dependents constituted the most affected group (32%, n = 375), whereas temporary workers were the least affected (4%, n = 47). Bite counts differed significantly across most demographic categories under an equal-distribution assumption (p < 0.0001), except gender. Males (49%) and females (47%) were bitten at similar frequencies (p = 0.63) (Table 2).

**Table 2:** Descriptive details of the bite-incidence from the secondary data recorded from 11 tea-estates in Udalguri district of Assam, India for the period 01 January 2023 to 31 May 2024.

Descriptive variables	Frequency (n = 1187)	p-value*
Age (years)*		
≤ 15	421 (35%)	<0.0001
16-30	300 (27%)	
31-45	252 (21%)	
46-60	153 (13%)	
>60	21 (1%)	
Missing data	40 (3%)	
Gender*		
Female	561 (47%)	0.63
Male	577 (49%)	
Missing data	49 (4%)	
Work status*		
Dependent	375 (32%)	<0.0001
Non-worker	307 (26%)	
Permanent	290 (24.5%)	
Temporary	47 (4%)	
Missing data @	168 (14%)	
**Vaccination dosage (PEP schedule)**		
Completed	511 (43%)	0.16
Incomplete	557 (47%)	
Missing data @	119 (10%)	
**Number of doses of PEP completed**		
Less than three	222 (19%)	<0.0001
Three or more	846(71%)	
Missing data @	119 (10%)	
Type of exposure*		
Bite	907 (76%)	<0.0001
Scratch	34 (3%)	
Missing data @	246 (21%)	
**Biting animal**		
Cat	147 (12%)	<0.0001
Dog	781 (66%)	
Others#	13 (1%)	
Missing data @	246 (21%)	

*Totals do not match because of missing data; #rat (3), rabbit (1), contact with rabies infected person/animals (9); *p-value of the chi-square test for goodness of fit when comparing with equal distribution between the levels; @was considered a category for the chi-square test.

### 3.3. Association tests

All 1,187 records were analysed for associations between the available predictor variables (gender, age, employment status) and outcomes (biting species, PEP completion, and ≥ 3 doses). Female victims were less likely than males to be bitten by dogs compared to other species (79% vs 88%) but were more likely to receive ≥ 3 PEP doses compared to less than three doses (Table 3). Children ≤ 15 years had higher odds of being bitten by dogs compared to other species, completing the full regimen, and receiving ≥ 3 doses compared with older individuals. Non-workers showed reduced odds of completing the PEP regimen, and along with temporary workers, were less likely to receive ≥ 3 doses compared to dependents.

**Table 3:** Test of univariable association between explanatory variables (age, gender and employment status) and outcomes (biting species, completion status of PEP, and number of PEP doses received) from the secondary data compiled from 11 tea-estates of Udalguri district in Assam, India.

	Biting species				PEP Regimen				Number of doses**			
**Gender (n)**	Dog	others	Odds ratio	p-value	Com	Inc	Odds ratio	p-value	≥ 3	<3	Odds ratio	p-value
Female (477)	375	102	0.5 (0.4-0.7)	**0.0002**	260	257	1.2 (0.9-1.5)	0.17	428	89	1.4 (1.1-1.9)	**0.01**
Male (457)	401	56	**Reference**		250	292	**Reference**		416	126	**Reference**	
**Age**												
≤15 years (343)	297	46	1.5 (1.1-2.3)	**0.02**	219	172	1.7 (1.3-2.1)	**<0.0001**	333	58	1.8 (1.3-2.6)	**0.0002**
>15 years (591)	477	114	**Reference**		290	381	**Reference**		508	163	**Reference**	
**Employment status**												
Non-worker (259)	217	42	0.8 (0.5-1.3)	0.3	128	173	0.4 (0.3-0.6)	**<0.0001**	212	89	0.3 (0.2-0.4)	**<0.0001**
Permanent (245)	198	47	0.6 (0.4-1.0)	0.06	180	136	0.8 (0.6-1.1)	0.1	240	41	0.7 (0.4-1.1)	0.1
Temporary (39)	33	6	0.8 (0.3-2.3)	0.6	24	22	0.6 (0.3-1.2)	0.1	34	12	0.3 (0.2-0.7)	**0.003**
Dependent (309)	269	40	**Reference**		180	105	**Reference**		255	30	**Reference**	

*Immunisation regimen was categorized as Complete (Com), if the schedule of 5 vaccines was followed, and Incomplete (Inc) if number of vaccines received was less than 5; ** < 3 – less than three vaccines administered; ≥ 3 –more or at least three vaccines administered.

### 3.4. Mixed effects logistic regression analysis and adjusted odd ratios

**Fig 2:**
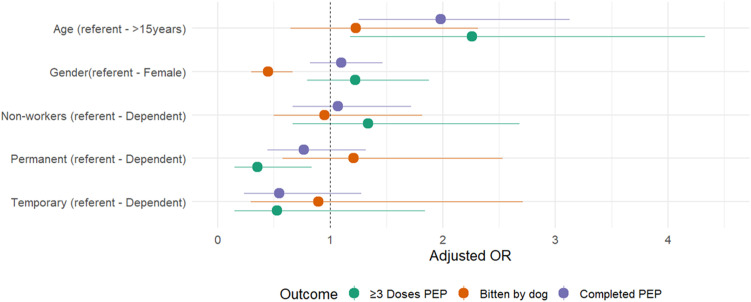
Depiction of the multivariable models and the adjusted odd ratios of combined regression of explanatory variables on the three outcomes.

A total of 824 entries that included all explanatory and outcome variables were considered to construct the generalised mixed effects logistic regression models. Model comparison and diagnostic statistics consistently supported the use of a generalised linear mixed-effects modelling framework over a standard generalised linear model. Across all three outcomes, inclusion of tea estate as a random effect substantially improved model fit and accounted for intra-estate clustering. For the biting species model, the AIC decreased from 716.2 (GLM) to 708.7 (GLMM), with the conditional R^2^ rising from 0.045 to 0.27, indicating that estate-level differences explained roughly a quarter of the total variance. For PEP completion*,* the AIC reduction was more pronounced (1140.2 to 1089.1), and the conditional R^2^ increased from 0.013 to 0.54, suggesting that over half of the variability in regimen completion was attributable to estate-level factors rather than individual covariates. The strongest clustering was observed for the ≥ 3 vaccine doses model, where AIC dropped sharply from 728.1 to 592.9 and the conditional R^2^ rose from 0.009 to 0.88, indicating dominant contextual influences at the estate level. Collectively, these metrics demonstrate that mixed-effects models more accurately captured the hierarchical structure of the data, with tea-estate-specific contexts exerting a substantial influence on all three outcomes. The multivariable models and the adjusted odds ratios are depicted in Fig 2).

**(a)** **Being bitten by a dog compared to other animals:** In the univariable analysis, females had lower odds than males of being bitten by a dog compared other species (OR = 0.4, 95% CI: 0.3–0.6; p = 0.0004). After adjusting for age and employment status in the multivariable GLMM, this association remained significant (aOR = 0.4, 95% CI: 0.3–0.7; p < 0.001). Neither age group nor employment status showed a significant association with the biting species. The random intercept variance for tea estate was 1.02, and the intra-class correlation coefficient (ICC) was 0.24 (Table 4).
**(b)** **Completing the PEP regimen**: In the univariable analysis, age and employment status were significant in univariable analysis, but only age remained so in multivariable models. However, in the mixed -effects model, after adjusting for gender and employment status, only age remained a significant predictor. Children aged ≤15 years had twice the odds of completing the vaccine regimen compared with adults (aOR = 2.0, 95% CI 1.2–3.1; p = 0.003). No strong evidence emerged to suggest an association between employment categories and completion of PEP after adjusting for covariates (Table 5). The random intercept variance was 3.7, with an intra-class correlation coefficient (ICC) of 0.53.
**(c)** **At least three doses of vaccines:** In the mixed-effects model, younger age and employment status were associated with receiving ≥3 vaccine doses. Children aged ≤15 years had higher adjusted odds (aOR = 2.2, 95% CI 1.2–4.3; p = 0.01), whereas permanent workers had lower odds (aOR = 0.3, 95% CI 0.1–0.8; p = 0.02) compared with dependents. The high random-effect variance (ICC = 0.88) (Table 6).

**Table 4:** Test of association between the explanatory variables (age, gender and employment status) and the biting species from the secondary data compiled from 11 tea-estates of Udalguri district in Assam, India using generalised mixed-effects regression models accounting for Tea-estate as random effect.

		Univariable				Multivariable				
Variable	Biting species - dog (%)	p-value	OR (95%CI)	Random variance	ICC	Coefficient (SE)	p-value	Adjusted OR	Random variance	ICC
**Constant (Intercept)**						-2.1 (0.5)				
Gender										
Female (419)	330 (81)	**0.0004**	0.4 (0.3-0.6)	0.9	0.22	-0.8 (0.2)	**<0.001**	0.4 (0.3-0.7)	1.02	0.24
Male (404)	361 (91)		**Reference**					**Reference**		
Age						0.2 (0.3)				
≤15 years (326)	282 (86)	0.1	1.4 (0.9-2.1)	0.9	0.22		0.5	1.2 (0.6-2.3)	1.02	0.24
>15 years (497)	409 (82)		**Reference**					**Reference**		
Employment status										
Non worker (251)	211 (84)	0.8	1.0 (0.6-1.7)	0.9	0.22	-0.05 (0.3)	0.8	0.9 (0.5-1.8)	1.02	0.24
Permanent (241)	194 (80)	0.1	1.5 (0.9-2.4)	0.9	0.22	0.2 (0.4)	0.6	1.2 (0.6-2.5)	1.02	0.24
Temporary (39)	33 (85)	0.8	1.1 (0.4-2.8)	0.9	0.22	-0.1 (0.6)	0.8	0.9 (0.3-2.7)	1.02	0.24
Dependent (292)	253 (87)		**Reference**					**Reference**		

OR: odds ratio; CI: confidence interval; SE: Standard Error; ICC: Intraclass Correlation Coefficient.

**Table 5:** Test of association between explanatory variables (age, gender and employment status) and PEP regimen completion from the secondary data compiled from 11 tea-estates of Udalguri district in Assam, India using generalised mixed-effects model accounting for Tea-estate as random effect.

		Univariable				Multivariable				
Variable	Completed regimen (%)	p-value	OR (95%CI)	Random variance	ICC	Coefficient (SE)	p-value	Adjusted OR	Random variance	ICC
Constant (Intercept)						0.8 (0.8)				
Gender										
Female (419)	221 (52)	**0.6**	1.1 (0.8-1.4)	4.0	0.55	0.1 (0.2)	0.5	1.1 (0.8-1.5)	3.7	0.53
Male (404)	203 (50)		Reference					Reference		
Age										
≤15 years (326)	187 (57)	**0.001**	1.7 (1.2-2.3)	3.8	0.55	0.7 (0.3)	**0.003**	2.0 (1.2 -3.1)	3.7	0.53
>15 years (497)	237 (48)		Reference					Reference		
Employment status										
Non worker (251)	117 (47)	**0.01**	1.6 (1.1-2.4)	3.6	0.52	0.3 (0.3)	0.7	1.1 (0.7-1.7)	3.7	0.53
Permanent (241)	123 (51)	0.09	1.4 (0.9-2.0)	3.6	0.52	-1.0 (0.4)	0.3	0.8 (0.4- 1.3)	3.7	0.53
Temporary (39)	21 (54)	0.9	1.0 (0.5-2.1)	3.6	0.52	-0.6 (0.6)	0.1	0.5 (0.2-1.3)	3.7	0.53
Dependent (292)	163 (56)		Reference					Reference		

The regimen was deemed complete if all 5 doses were received. OR: odds ratio, CI: confidence interval, SE: Standard Error; ICC: Intraclass Correlation Coefficient.

**Table 6:** Test of association between explanatory variables (age, gender and employment status) and more or at least three vaccines received from the secondary data compiled from 11 tea-estates of Udalguri district in Assam, India using generalized mixed-effects model accounting for Tea-estate as random effect.

		Univariable				Multivariable				
Variable	≥3 doses (%)	p-value	OR (95%CI)	Random variance	ICC	Coefficient (SE)	p-value	Adjusted OR	Random variance	ICC
Constant (Intercept)						-0.8 (0.8)				
Gender										
Female (419)	355 (85)	0.3	1.3 (0.8-1.9)	26.3	0.9	0.2 (0.2)	0.3	1.2 (0.8-1.8)	24.4	0.88
Male (404)	328 (81)		Reference					Reference		
Age										
≤15 years (326)	286 (87)	0.08	1.5 (0.9-2.4)	25.5	0.9	0.8 (0.3)	**0.01**	2.2 (1.2-4.3)	24.4	0.88
>15 years (497)	397 (80)		Reference					Reference		
Employment status										
Non worker (251)	183 (73)	**0.003**	2.3 (1.3-3.9)	23.5	0.9	0.3 (0.3)	0.4	1.3 (0.7-2.7)	24.4	0.88
Permanent (241)	211 (87)	0.3	0.7 (0.4-1.4)	23.5	0.9	-1.0 (0.4)	**0.02**	0.3 (0.1-0.8)	24.4	0.88
Temporary (39)	30 (77)	0.9	1.1 (0.3- 3.3)	23.5	0.9	-0.6 (0.6)	0.3	0.5 (0.1-1.8)	24.4	0.88
Dependent (292)	259 (89)		Reference					Reference		

OR: odds ratio, CI: confidence interval, SE: Standard Error, ICC: Intraclass Correlation Coefficient.

### 3.5. Distribution of bites and employment status

Dependents had the highest odds of getting bitten. Compared with dependents, non-workers (OR 0.5, 95%CI 0.4-0.7, p < 0.0001), and temporary workers (OR 0.2 95%CI 0.1 -0.3, p < 0.0001) had significantly lower odds, while permanent staff did not differ significantly.

### 3.6. Monthly and seasonal variation in bite incidence

**Fig 3:**
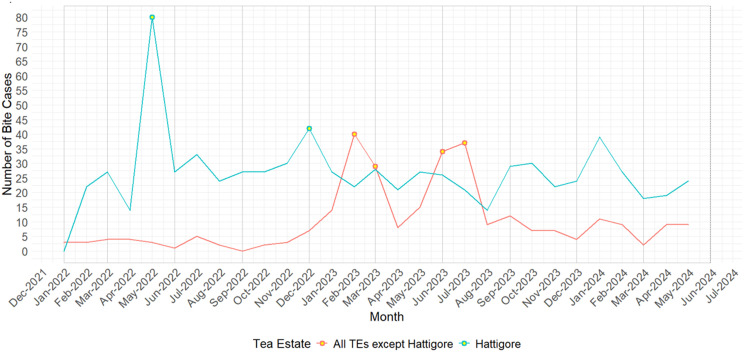
The line graph depicting the monthly trend of bites recorded for 2022-23 from the secondary data collected from Hattigore and the sum of all other TE available with the estate’s dispensaries and hospitals. The highlighted points are peaks of bite cases above the respective means.

The monthly distribution of bite cases across TEs is shown in Fig 3. Hattigore TE consistently reported higher bite counts than the combined total of all other TEs. Spikes were observed during winter (Dec–Feb) in both 2023 and 2024, with a secondary rise during the monsoon (Jun–Aug). Post-monsoon months (Sep–Nov) showed a contrasting decline. Weak negative correlations were observed between monthly bite counts and average temperature (r = –0.18) and humidity (r = –0.20).

## 4. Discussion

### 4.1. Overview

Tea-estate -communities face unique health challenges including rabies-related deaths resulting from animal bites. This study, the first in this domain comprehensively evaluates incidences of animal bites in the TE community in a district of Assam state in India. It demonstrates high vulnerabilities to potential dog-mediated rabies and highlights the disparities in adherence to preventive practices attributable to spatial, demographic and behavioural community practices, while underscoring the need for robust animal bite reporting systems that encompasses One Health programming and aggressive rabies awareness campaigns for vulnerable communities such as TE workers.

A markedly high annual bite incidence rate (36.9/1000 individuals) was reported in Hattigiore, TE, more than twice the overall annual incidence rate (16.0/1000 individuals). A consistently elevated monthly bite number (Fig 3) suggests the presence of a persistent risk due to possibly a high FRD population supported by inefficient waste management similar to other rural and semi-rural milieu of the country. A better reporting and record-keeping in Hattigore TE, compared to other sites, where records of bite care were poorly maintained is also a reason for a comparative high incidence along with the facilitation of easy and no-cost availability of PEP by the TE administration, as anecdotally corroborated by the estate welfare manager. While the availability of PEP in one TE promotes better record maintenance, it emphasizes the challenges of uniform provision of prophylaxis across other TEs. A structured bite-reporting system linked with PEP uptake is recommended for all TEs to help ascertain the true prevalence of bite [33]. The variability of reporting, and the proclivity of TE workers to avoid seeking medical assistance following a bite could also be due to variability in the intensity of sensitising the worker communities to compulsorily report exposures, even if it is just a scratch. It was evident from records at Hattigore and Dimakuchi TEs that the number of scratches reported was comparatively higher than in other estates. Enhancing sensitivity about fatal consequences of ignored dog-bite exposures has shown improved reporting [34–37].

Elevated annual incidence in Dimakuchi (26.5/1000 individuals), Paneery (14.7/1000 individuals), and Bhutiachang (11.6/1000 individuals) is likely attributable to the high density of FRD closer to urban centre, which support a greater carrying capacity due to dense human populations, as compared to TE along national and international borders that have sparse human habitation [38]. Interestingly, Corramore TE, located along the district periphery, also exhibited high incidence (15.1/1,000), contradicting the assumption that remoteness from human habitats implies reduced risk. This trend may be explained by the transboundary movement of FRDs, especially given Corramore’s proximity to the international border with Bhutan [39,40]. These findings highlight the need for systematic studies assessing FRD movement and densities in border regions to elucidate spatial heterogeneities in bite risk. The high incidence among permanent workers (19.0/1000 individuals per year) may be attributed to the outdoor nature of their work, which requires frequent movements and prolonged interaction with environments shared by FRD, thereby increasing their vulnerability [41]. In contrast, the similar high incidence among the ‘dependent’ category - which includes comprises children, housewives, and the elderly may be explained by multiple factors. While an increased exposure of children and elderly is plausible, this category may also have a higher likelihood if reporting bite cases compared to other groups.

The disproportionate representation of children aged ≤15 (37%) is consistent with previous studies globally and nationally. Children are particularly vulnerable to dog bites due to their spontaneous affection for animals, smaller stature and lack of awareness about safe interactions [42,43]. However, a significant concern is that 38% of these children are under 5 years’ age, including a 9-month-old baby. One likely reason for such a high proportion of young children exposed to animal bites is the lack of supervision when parents leave for work [44]. Many of these bites, which could potentially result in rabies, may go unreported [44–48]. Introducing creche facilities in TE could help reduce dog bites among young children by ensuring supervision.

### 4.2. Determinants of bite exposure and PEP adherence

Significant associations emerged between the demographic variables (age and gender) with exposure to dog bites and adherence to PEP (Table 3). When accounting for intra-estate clustering using mixed-effects logistics regression, gender and age emerged as significant predictors of exposure and PEP adherence. A moderate to substantial TE level heterogeneity indicates strong estate-oriented context in rabies prevention strategies (Tables 4–6). Higher exposure of males to bites by dogs can be due to the heightened exposure to dogs because of outdoor work patterns [49]. A moderate clustering effect (ICC = 0.24) indicated estate level variations such as dog population density and waste-management influencing dog-human interactions [50,51]. While females and children aged ≤ 15 years and non-workers show higher odds of receiving post-bite PEP, lack of adherence in males, permanent employees, and dependents reflect potential barriers and differences in healthcare access or compliance [11,52].

The low completion rate among the ‘dependents’ may be due to the financial burden faced by parents or heads of families of losing daily wages to accompany children or other dependents to the hospital for PEP. Nonetheless, the higher odds of children <15 years for receiving >3 doses is a contrasting finding and hence, the speculation of parents’ apprehension of losing wages warrants further investigation. On the contrary, ‘non-workers’ are also at higher odds to receive at least three doses of vaccines than ‘dependent’ counterparts. It is a surprising finding because, as opposed to the ‘non-workers’, the ‘dependents’ are provided the vaccines free of cost and adherence to PEP generally improves when supplied at reduced or no cost [10,53]. However, Hattigore TE is an exception where PEP was free even to the ‘non-workers’, explaining the increased overall odds. The ‘non-workers’ category includes the unemployed population which may have greater flexibility in attending vaccination appointments without the concern of wage loss, hence the high odds compared to other categories. Alternatively, this category may be employed in informal or off-estate employment potentially earning more than tea -estate workers thus being better able to pay for the vaccination costs. However, these reasonings remain speculative and call for exploratory studies to shed more light on the motivation of ‘non-workers’ in this context.

The ‘age’ variable is an important determinant affecting PEP regimen compliance or receiving at least three doses, especially when adjusted for gender and employment status. This finding emphasizes the need for age-specific interventions, such as targeted health messaging and strategies promoting PEP adherence to ensure that children and their caregivers are adequately informed about rabies prevention and management. The demographic variables especially the ‘employment status’ evinced nuanced interdependencies post multivariable analyses adjustment. A structured investigation such as questionnaire surveys on awareness about rabies and post dog-bite practices are recommended to further explore the mutual interdependence of socio-demographic variables influencing exposure to dog bites by using multivariable framework.

### 4.3. Estate-level clustering and model implications

The use of mixed effects models appears justified as it revealed moderate (ICC = 0.24) to strong clustering effect (ICC = 0.88). While moderate clustering suggests influence of estate-level factors like dog density, the high ICC for PEP completion underscores contextual barriers such as health care access, counselling quality or vaccine availability among the different TE. Further, a very high clustering effect indicates that estate-specific contextual factors such as location of vaccination points, and health care facilitation, and awareness enhancing measures strongly influence adherence to PEP. These findings demonstrate that more than individual influences, determinants at the estate level that fall in the domain of the estate management substantially shape the PEP completion rates. It is recommended that the interventions aimed at rabies elimination in TE settings should incorporate both individual- and system-level components: targeted communication for adult men and workers, flexible vaccination delivery mechanisms that accommodate labour schedules, and estate-specific micro-planning to address local barriers.

### 4.4. Seasonality of bites in tea-estates

Although no clear seasonal trend emerged, notable spikes during the winter (Dec- Feb) and monsoon (Jun-Aug) seasons suggest changes in canine activity which presumably increases the bite risk. Nonetheless, the potential utility of seasonally tailored community intervention programs, enhanced surveillance during high-risk periods, and timely rabies vaccination drives in canines and human populations prior to high-risk periods cannot be undermined [54]. As the data were collected from only a few estates in a single district of Assam, a key limitation of this study, a further exploration of seasonality of dog-bites is recommended [55].

### 4.5. Operational barriers and health seeking behaviour

The informal interactions with the health staff at a TE during the data collection revealed, a persistent challenge highlighting inconsistent availability of PEP in healthcare facilities. Bite victims are often directed to private pharmacies, where costs and travel distances become deterrents to completing the full regimen. Similar barriers such as remoteness and limited vaccine availability have been widely cited in many studies [56–58]. The recent recommended shift to a reduced dosage of PEP administered intradermally instead of the intramuscular route could be a solution to economise PEP usage in such high-risk communities [10,11,59]. However, feasibility studies considering bite severity and injury categories are necessary before broader implementation. Another concern raised by healthcare staff is the reliance on traditional healers resulting in underreporting and non-completion of PEP. Engaging the traditional healers through citizen science initiatives may enhance case reporting and even encourage timely referral to healthcare facilities, an initiative [60–62]. Such strategies, though speculative, hold promise and warrant field-testing.

A strong anecdotal perception persists in the TE that the number of FRD, semi-owned and feral, is high among communities, and residents are unaware of anti-rabies immunisation in dogs. Tea-estates are rarely included in rabies awareness campaigns, and no Animal Birth Control (ABC) programs are rarely conducted. Such interventions are cardinal to prevent and control dog-mediated rabies. We recommend initiating these interventions along with need assessment studies to tailor interventions to the specific needs of TE communities [63–65].

### 4.6. Limitations and future directions

Although limited by retrospective nature and deficient data due to weak bite recording mechanisms in the TE, this study highlights the need for a comprehensive, focused, demographically sensitive approach to rabies prevention in the TE communities. We recommend investigative research exploring the factors influencing the high incidence of dog-mediated rabies in such vulnerable communities, as few such studies exist. The likely logistical barriers hindering PEP access to healthcare facilities must be removed [58,66]. At the same time, rabies awareness and relevance to One Health need emphasis to devise robust strategies that reduce dog bites and potential rabies deaths [67–69]. Educational campaigns should be prioritised for vulnerable populations, caregivers and parents [67]. A robust dog-bite surveillance system sensitive to spatial and temporal trends is recommended [70,71].

These findings can be a harbinger for future research to expand its geographic scope to include a broader representation of tea -estate communities across Assam and other parts of India. Mixed-method approaches including household surveys, structured interviews with tea -estate workers, and focus group discussions should be employed to uncover the socio-economic determinants and behavioural patterns contributing to high dog-bite incidence and poor PEP adherence. Studies focusing on the ecology of FRD populations, their density, movement, and interactions with tea -estate residents can help design more effective ABC and vaccination interventions. Socio-economic variables, such as household income, rabies-related awareness, and access to healthcare, should be systematically explored to identify barriers and facilitators of rabies control. Additionally, longitudinal studies are needed to assess seasonal variations in bite incidents and evaluate the long-term impact of community-based interventions like rabies education drives and child-safe creche facilities. This study lays the foundation for deeper inquiry into the vulnerability of tea -estate communities to dog-mediated rabies and the development of integrated, One Health-aligned solutions to mitigate this public health burden.

## Supporting information

S1 Data Excel sheet with tea -estate retrospective data_Udalguri. (XLSX)
